# Resistance to innovation in low-income populations: The case of university students' resistance to using digital productivity applications

**DOI:** 10.3389/fpsyg.2022.961589

**Published:** 2022-10-07

**Authors:** Mohammad Alshallaqi, Hussam Al Halbusi, Mazhar Abbas, Homoud Alhaidan

**Affiliations:** ^1^Department of Management and Information Systems, College of Business Administration, University of Hail, Ha'il, Saudi Arabia; ^2^Ahmed Bin Mohammed Military College, Al Rayyan, Qatar

**Keywords:** consumer resistance, innovation resistance theory, adoption of innovation, resistance to innovation, consumer behavior, consumers' characteristics

## Abstract

Innovation resistance research remains in its early stages. Efforts to define and comprehend consumer resistance to innovation necessitate in-depth studies that consider the contextual factors of resistance to innovation. To address this challenge, this research explored consumer resistance to innovation in a low-income population, namely, university students on financial support. The innovation under this study is the productivity applications provided for free by the University of Hail, Saudi Arabia, to all students. This study explores variables such as value barrier, risk barrier, tradition barrier, and image barrier and how they impact consumer resistance to innovation in a low-income population. We extend the theory by investigating the moderating roles of consumer characteristics (motivation, self-efficacy, emotion, and attitude toward existing products). The current study is based on an online survey of low-income students at a Saudi Arabian University; 258 cases were gathered. We found that all the direct effects of the variables (i.e., Value Barrier, Risk Barrier, Tradition Barrier, and Image Barrier) were positively related to consumer resistance to innovation. Importantly, consumer characteristics significantly moderate this relationship, as the relationship is stronger when the measurements for consumer characteristics are high.

## Introduction

Companies and governments around the world invest billions of dollars in research and development to produce innovative products and services (Pérez-Luño et al., 2019). However, the question remains: What happens when consumers resist these innovations? The result is that significant investments are lost. Therefore, it is vitally important to research and understand why and how consumers resist innovations and how to design products and services that consumers will not resist. In this pursuit, an emerging research field called *consumer resistance to innovation* has been developed to investigate the factors that cause such resistance (Ram and Sheth, 1989; Fattah et al., 2021). This study investigates barriers such as value, risk, tradition, and image, and the moderating role of consumer characteristics in resistance to innovation (Pelau et al., 2021).

Ram and Sheth (1989) established the resistance to innovation model, which is based on the characteristics of consumers as well as innovation, and explained why consumers are unable to adopt innovations. Moreover, Ram and Sheth (1989) found that consumers are resistant to innovation because of difficulties in implementing change, together with the ensuing arguments. For consumers, these issues can be divided into psychological and practical barriers. Individuals are unable to accept novel concepts and products because of psychological obstacles, such as image and tradition barriers (Naveed et al., 2022; Zhang et al., 2022). Similarly, a consumer's belief system is often the source of psychological barriers (Ram and Sheth, 1989). Furthermore, other aspects, such as motivation, personality, perception, behavior, attitude, prior experiences with innovations, norms, and users' belief systems, can all be sources of psychological barriers (Ghazali et al., 2022; Li et al., 2022).

With the development of these variables, research on consumer resistance to innovation has proliferated. Researchers have examined a wide variety of innovations in different settings and among different population segments (see Talwar et al., 2020). In their extensive review of the literature, Talwar et al. (2020) found that digital innovations remain understudied in terms of consumer resistance to innovation. Additionally, while the research on this topic spans a wide variety of populations, low-income populations are largely overlooked. This issue of resistance to innovation in low-income populations, therefore, points researchers in this new direction. This study investigates resistance to digital innovation (office productivity applications) among a particular low-income population, namely, university students on financial support.

Little is known about the key variables that impact consumer resistance to innovation, especially in low-income populations. This study aims to analyze the most relevant aspects that affect low-income consumers' resistance to innovation (Uzir et al., 2020). The research focused on examining the link between functional and psychological barriers and consumer resistance to innovation and how consumer characteristics might moderate this relationship (Citrin et al., 2000; Ho and Wu, 2011; Kunz et al., 2011; Chao et al., 2012). Baron and Kenny (1986) proposed using the moderator variable if the associations between the dependent and independent variables were not sufficiently established as either inconsistent/insignificant or consistent/significant. In this regard, consumer characteristics were used as moderators.

The study focused on a particular low-income population, namely, undergraduate university students on financial support in a Saudi Arabian University. Undergraduate students on financial support receive monthly stipends from the government to help them with their university and life expenses. This fact characterizes them as a low-income population. Additionally, the study focused on a specific digital innovation, namely, productivity applications that aim to help students manage their time and tasks in an efficient and streamlined manner. These applications are provided for free by the university to all students to help them manage their studies effectively (Alnoor et al., 2022).

The paper is structured as follows. First, we explore the literature on resistance to innovation in greater depth to develop our hypothesis. Then, the research framework is presented, along with the study's hypotheses. After an exposition of the study's research methodology, the results are presented, followed by a discussion that explains the contributions of the study. The paper concludes with a section that summarizes its results, identifies its limitations, and puts forth future research opportunities.

## Literature review

### Consumer resistance to innovation

Innovation resistance research is an important field of study for understanding the reasons behind consumers' rejection of technology and, hence, the failure of such technological artifacts. In this pursuit, researchers have sought to investigate and explore the myriad variables that might, in some form or other, lead consumers to resist an innovation (Cornescu and Adam, 2013; Fattah et al., 2021). For example, Mohtar et al. (2015) argued that personal beliefs and cultural norms play a vital role in consumer resistance to innovation. Similarly, Cornescu and Adam (2013) argued that resistance attitudes play a key role in fostering resistance to innovation. It is also important to consider changes in consumption patterns and how they affect consumer resistance to innovation (Gatignon and Robertson, 1991). Such changes in consumption patterns include, but are not limited to, changes in lifestyle and standards of living (Watson, 1971; Zaltman and Duncan, 1977). Additionally, it has been noted that consumers' excitement toward an innovation plays a key role in whether they would resist an innovation (Gold, 1981; Murdock and Franz, 1983; Salerno, 1985). In other words, the lack of excitement toward innovation can be reinterpreted as a form of resistance to that innovation (O'Connor et al., 1990; Ellen et al., 1991).

Few studies have addressed innovation resistance with regard to digital services uptake (Brehm, 1966; Mohtar and Abbas, 2015). Stressing consumer resistance is critical because consumer perception of products plays a vital role in resistance to innovation. As a result, consumer resistance to innovation is crucial because of its positive and negative repercussions, such as the adoption of innovations or the lack thereof (Mohtar et al., 2015; Yu et al., 2015; Andronie et al., 2021). Furthermore, while few studies on consumer resistance to innovation (e.g., Leonard, 2004; Mohtar and Abbas, 2015) have specified factors that positively affected such resistance, some factors remain unexplored (Hoong et al., 2019; Al Halbusi et al., 2022). As a result, to fill a gap in the knowledge, this study aimed to identify the most important determinants of consumer resistance to innovation, with specific attention to low-income populations. In this pursuit, there are few studies that illustrate the relationship between consumer barrier variables (i.e., value barrier, risk barrier, tradition barrier, and image barrier) and consumer resistance to innovation.

Conversely, the degree of consumer resistance is determined by how consumers perceive innovation. The most important component of innovation resistance is the consumer's characteristics and attributes. Variety seekers or innovators like inventing for the sake of new experiences (Barbu et al., 2021); therefore, they are less resistant to new items. Self-efficacy, for example, is a personality feature that influences how consumers react to new products. In the case of innovations, for example, this cannot be verified prior to purchase. A consumer with poor self-efficacy would be more likely to wait until the product's performance has been satisfactorily shown. As a result, consumer resistance to innovation is inversely correlated with self-efficacy (Rokeach, 1973; Hassan et al., 2020).

Motivation is one of the predictors or drivers of consumer resistance to innovation. Consumer buying behavior that is dependent and shaped by ingrained “habits” (Sheth, 1981) is known to cause resistance to innovation. If consumers are content with their current routine and the innovation threatens to disrupt that routine, they are likely to reject the change. As a result, consumer resistance to innovation has a negative association with motivation. Similarly, resistance is influenced by the consumer's receptive attitude toward innovation. Consumers' positive attitudes regarding existing innovation lead to increased resistance toward proposed or new innovative products or technologies (Al Halbusi et al., 2021). If consumers do not feel the need for innovation, they are likely to oppose it (Davis et al., 1992; Lee et al., 2007). Furthermore, resistance to innovation is likely if the consumer's impression of the invention remains positive both before and after it is implemented.

Risk, use, and value barriers, often known as *functional barriers*, prevent people from embracing new ideas. These functional barriers may develop, for example, if consumers notice major changes as a result of adopting an innovative product (Ram and Sheth, 1989). In addition, resistance to innovation theory incorporates innovative features, such as relative advantage, better product adoption, complexity, and perceived risk, as elements affecting technology acceptance or key grounds for rejection. Likewise, the degree of consumer resistance is determined by the characteristics of innovation as perceived by consumers. Ram and Sheth (1989) also supported Rogers's (2003) assertion that innovation features, such as comparative advantages over an innovation, may be seen from the standpoint of economic benefits or cost savings. In addition to increasing value, innovation may offer superior performance at comparatively cheap prices. Consumers are more likely to oppose innovation if it has a poor competitive advantage over already accessible substitutes. Furthermore, perceived risk is linked to innovation uptake. The perceived amount of risk varies depending on the type of innovation. Consumers regard continual or small improvements as having a lower perceived risk (Gatignon and Robertson, 1991).

Intermittent or large innovations, on the other hand, pose a danger of disrupting consumer habitual behavior; the bigger the perceived risk, the greater the resistance to innovation. The degree to which the invention is seen as somewhat difficult to understand and operate is another aspect of innovation. It might be complicated or tough to be an early adopter or prospective early adopter of some inventions or fresh concepts. Because the phrase *adoption rate* implies that innovation can be adopted or rejected, the price of the innovation can also be a predictor of the rate of adoption (Abbas et al., 2017). This means that when the cost of new items is high, the rate of adoption falls, thus increasing consumer resistance to innovation (Rogers et al., 2014). Consumers' inventiveness is also based on innovation theory. According to Rogers and Shoemaker (1971), consumer innovativeness is defined as the degree to which consumers are early adopters of innovation compared to their social group. Consumers with a high level of innovativeness are defined by Blackwell et al. (2006) as being ready to make changes in goods and ideas (see also Bartels and Reinders, 2011).

As a result, Ram and Sheth (1989) proposed that rejection is the most powerful type of consumer resistance to innovation, as opposed to other results, such as postponement and delay, which are primarily influenced by situational or innovation variables. For instance, product perceived difficulties influence whether an innovation is adopted or rejected. Furthermore, Yadav and Varadarajan (2005) found that consumer rejection is a critical predictor of resistance to innovation by consumers. In this study, we examined the hurdles that may cause resistance to innovation. There were a variety of barriers to consider; however, the research concentrated on the barriers of value, risk, tradition, and image. In addition, the research examined the moderating role. Furthermore, consumer characteristics, such as motivation, self-efficacy, emotion, and attitude, were investigated as moderators.

## Research model and hypothesis development

### Value barriers

Value barriers stem from a mismatch with preexisting values, especially with regard to weighing the cost of adoption and innovation against the invention's benefits (Aggarwal and Prasad, 1998; Lian and Yen, 2013). To possess low-value barriers, innovations must deliver greater value to users in exchange for their labor in learning and adapting to such systems (Kliestik et al., 2022).

User behavior influences the resistance, adoption, and usage of innovations. Most previous research suggests that there is a negative association between value barriers and user intentions in diverse settings, such as shopping online (Lian and Yen, 2013; Uzir et al., 2021a), mobile shopping (Moorthy et al., 2017), and mobile services (Joachim et al., 2018). User resistance to m-banking (Yu and Chantatub, 2015) and e-tourism has also been connected to value barriers (Jansukpum and Kettem, 2015). For instance, Sivathanu (2018) observed a positive correlation between value barriers and user resistance.

**Hypothesis 1:** Value barriers have a positive effect on consumer resistance to innovation.

### Risk barriers

Risk barriers relate to the resistance that occurs due to uncertainties, which are an unavoidable part of every innovative product. Dunphy and Herbig (1995) and Aldás-Manzano et al. (2009) argued that an innovation's acceptance is determined by the degree of uncertainty it causes. Ram and Sheth (1989) identified four categories of risk: physical, economic, functional, and social. With respect to mobile banking, for instance, users may be at risk of fraud, money loss, poor Internet access, or dwindling smartphone battery life. Risk barriers have already been found to have an adverse influence on users' intentions and behavior in the research. Risk barriers, for example, have a negative effect on usage intention in a multitude of fields, such as commerce conducted through mobile phones (Al-Jabri and Sohail, 2012; Moorthy et al., 2017), shopping using the internet (Lian and Yen, 2013; Lian et al., 2013), using mobile phone applications to shop for new products (Gupta and Arora, 2017), m-banking as a medium for banking service delivery (Laukkanen, 2016), and mobile gaming (Moorthy et al., 2017). In other words, when risk barriers become high, they result in unfavorable user behaviors, such as resistance. Therefore, prior research has revealed that resistance to various digitization programs, such as m-banking (Yu and Chantatub, 2015) and e-tourism, results in greater risk barriers (Jansukpum and Kettem, 2015).

Risk barriers may become prospective hurdles to mobile online applications' acceptance, usage, and plans to recommend them as a result of the uncertainties they bring. Concerns about safety, confidentiality, and trust abound with mobile applications (Marett et al., 2015; Uzir et al., 2021b; Hassan et al., 2022). Two such concerns are the loss of sensitive information and the creation of security breaches (Ediriweera and Wiewiora, 2021). According to the literature, risk barriers have a negative correlation with a user's intention to utilize and embrace digital services (El-Haddadeh, 2020). A lack of knowledge of the security and privacy consequences of digitalized services among potential and current users might lead to the establishment of barriers related to risk (Luo et al., 2010; Ullah et al., 2021).

**Hypothesis 2:** Risk barriers have a positive effect on consumer resistance to innovation.

### Tradition barriers

Any product or service's success is generally influenced by traditions that guide a user's behavior. Scholars say that traditions are profoundly embedded in the lives of people and that any potential conflict with them results in a significant consumer reaction in the form of unfavorable public shaming and ostracization (Kaur et al., 2020). The impediments that any innovation produces when it disturbs a user's established habit, culture, or behavior are known as *tradition barriers* (Kumar et al., 2022). Adoption hopes for any new breakthrough are also hampered by traditional restrictions (Antioco and Kleijnen, 2010).

Tradition barriers are linked to mobile application resistance (Jansukpum and Kettem, 2015; Yu et al., 2015). The adoption of mobile payments in India, for instance, has resulted in a significant shift in how consumers make payments. Traditionally, consumers used cash to make payments, but today, they utilize mobile devices to make cashless purchases (Patil et al., 2020). Furthermore, a previous study looking at the adoption of mobile payment applications in India immediately after the demonetization crisis in November 2016 discovered that user resistance to such applications is linked to tradition barriers (Sivathanu, 2018; Mishra et al., 2022).

**Hypothesis 3:** Tradition barriers have a positive effect on consumer resistance to innovation.

### Image barriers

Image barriers refer to the identity associated with an innovation. Such identities might stem from different sources, such as the innovation's country of manufacture, the cultural logic that surrounds a specific innovation, or even the way it is marketed and communicated to the public (Ram and Sheth, 1989). Image barriers are used to combat a negative view of innovation as a result of the presumed amount of complexity associated with its use (Lian and Yen, 2013). Consumers, for example, rarely consider mobile applications secure, resulting in a poor image (Kaur et al., 2020). According to a prior study, when it comes to numerous digitization activities, image is a barrier that has a negative influence on consumers' behavior. For example, image is adversely connected with users' adoption-related intentions toward mobile applications (Laukkanen, 2016; Joachim et al., 2018). Furthermore, client resistance to mobile banking is caused by image barriers (Yu and Chantatub, 2015).

**Hypothesis 4:** Image barriers have a positive effect on consumer resistance to innovation.

### Consumer characteristics

It is critical to assess the impact of the characteristics of both the consumer and the innovation on consumer resistance to innovation because these characteristics have been proven to be determining factors for such resistance (Tornatzky and Klein, 1982; Gatignon and Robertson, 1991; Veryzer, 1998). Additionally, it has been shown that resistance to innovation is dependent on the consumer in that it changes from consumer to consumer, further buttressing the importance of studying consumer characteristics (Ram and Sheth, 1989). The same can also be said about the innovation itself, in that the characteristics of the innovation affect whether consumers resist it (Barczak et al., 1997; Wang et al., 2003).

**Hypothesis 5:** Consumer characteristics moderate the relationship between value barriers and consumer resistance to innovation, such that the relationship is stronger when the measurements for consumer characteristics are high.
**Hypothesis 6:** Consumer characteristics moderate the relationship between risk barriers and consumer resistance to innovation, such that the relationship is stronger when consumer characteristics are high.
**Hypothesis 7:** Consumer characteristics moderate the relationship between traditional barriers and consumer resistance to innovation, such that the relationship is stronger when consumer characteristics are high.
**Hypothesis 8:** Consumer characteristics moderate the relationship between image barriers and consumer resistance to innovation, such that the relationship is stronger when consumer characteristics are high.

## Methods, sample size, and procedure

The respondents for this study were students on financial support studying at the College of Business Administration, University of Hail. The total population size was 1,800 low-income students. With respect to specifying an appropriate sample that could yield reliable results for the study, we chose this particular sample, as most students were taking courses related to business and innovation and were receiving financial support from the university. Poverty is a consequence of the digital divide, which is the disparity in access to and use of technology and the Internet. Digital resources are more likely to be accessible to the well-educated and affluent, whereas nonwhites and people of lower income are more likely to lack access to them (Kezar et al., 2022). Therefore, a specific digital innovation such as productivity applications in this case aimed at helping students in managing their time and tasks in an efficient and streamlined manner. Thus, they were the best sample for this research.

It was crucial to have a sample size that was precise and adequate. Therefore, Hair et al. (2011) suggested that an appropriate sample size conducive to statistical analysis must be at least 10–20 times greater than the required variables. Before we circulated the survey, the registrar of the college was approached to ask permission. Once permission was granted, the online survey was distributed through emails and WhatsApp groups. Thus, out of 280 distributed questionnaires, 258 responses were returned. Uncompleted responses were excluded, and data from 258 were used for further analysis. This resulted in a response rate of 92%. Among the respondents, 67.4% were men, and 33.56% were women. The age of 60% of the respondents was 18–24 years old, followed by 25–29 (9.2%) and 30–39 (30.8%) years old.

### Measurement of the variables

All measures were derived from prior reliable studies. Before the beginning of the data collection stage, the questionnaire was verified by three academic experts in related fields. After the English version was verified, the items were translated from the English version to the Arabic version because the targeted respondents were native Arabic speakers. The translation process was based on the double-blinded principle, where the original English version of the scales was translated into Arabic, and the Arabic version was back-translated by two professional researchers (Brislin, 1970) to assure their validity.

We measured the Value barrier (VB) with 2 items that were slightly adapted from Laukkanen (2016). The Risk barrier (RB) was assessed with 4 items taken from Laukkanen (2016). The Tradition barrier (TB) was measured with 5 items adapted from Laukkanen (2016). To measure the Image barrier (IB), 2 items were slightly adapted from past studies (Laukkanen, 2016). Consumer characteristics were measured with subdimensions involving attitude toward existing products (three items), motivation (four items), social influence (five items), and emotions (six items). These items were taken from Schwartz and Sagiv (1995), Richins (1997), Wang et al. (2003), Walczuch and Lundgren (2004), Agosto and Hughes-Hassell (2005), Reynolds et al. (2006), Park and Chen (2007), and Carayannis et al. (2013). Finally, consumer resistance to innovation was evaluated with seven items adapted from Sheth (1981), Szmigin and Foxall (1998), and Yang (2005). All measures were assessed on a 5-point Likert scale (1 = Strongly Disagree and 5 = Strongly Agree).

## Data analysis and results

For several reasons, structural equation modeling (SEM) using partial least squares (PLS) with the Smart PLS 3.3.3 software (Ringle et al., 2005) was used to evaluate the given hypotheses. This thorough, rigorous, and systematic technique is suitable for complex causal analyses, including first- and second-order constructs, and it does not require strict assumptions regarding the underpinning variables (Henseler and Sarstedt, 2013; Hair et al., 2017). Additionally, we used the 5,000-subsample approach to construct bootstrap t-statistics with n-1 degree of freedom to test the significance of the path coefficients (where n is the number of subsamples).

### Common method bias

As the independent and dependent variables were gathered using the same questionnaire, the issue of common method bias (CMB) might have occurred. To address this issue, we adopted a two-pronged strategy of procedural and statistical techniques (Podsakoff et al., 2012; Tehseen et al., 2017). On a procedural level, we employed numerous measurement scales in the survey instrument. We also reminded respondents that there were no correct or incorrect responses and that their names would be considered anonymous.

In terms of statistics, we used two methods: Harman's single-factor analysis and the complete collinearity test, depending on Variance Inflation Factors (VIFs). The findings of Harman's single-factor analysis indicated that a single component accounted for just 13.82% of the overall variance. Second, we employed VIFs to conduct a thorough collinearity test (Kock, 2015). (Kock and Lynn, 2012) advised performing such a test to assess both vertical and lateral collinearity. According to (Kock and Lynn, 2012), when the VIF is more than 3.3, it implies pathological collinearity, implying that the model is affected by Common Method Variance (CMV). Nevertheless, as indicated in Table 1, this trial was CMV-free.

**Table 1:** Common method variance assessment via full collinearity estimate criteria.

**Variable**	**Value barrier (VB)**	**Risk barrier (RB)**	**Tradition barrier (TB)**	**Image barrier (IB)**	**Customer characteristics**	**Customer resistance to innovation**
VIF	1.704	1.541	2.410	1.267	1.137	2.215

VIF, variance inflation factor.

### Confirmatory factor analysis

Before attempting to utilize a structural model, its properties (item reliability, internal consistency reliability, convergent validity, and discriminant validity) had to be demonstrated (Hair et al., 2017). Table 2 shows that the bulk of the items scored greater than the 0.707 criterion (Hair et al., 2017, 2019). Cronbach's alpha and composite reliability were employed to assess the internal consistency of the constructs. Table 2 shows that both strategies produced good results that were greater than the cutoff of 0.70 (Hair et al., 2017, 2019). In terms of convergent validity, the average variance extracted (AVE) was likewise above the 0.5 criterion (Hair et al., 2017, 2019) (see Table 2).

**Table 2:** Measurement model, loading, construct reliability, and convergent validity.

**First-order constructs**	**Second-order constructs**	**Items**	**Loading (>0.5)**	**CA (>0.7)**	**CR (>0.7)**	**AVE (>0.5)**
Value barrier (VB)		VB-1	0.738	0.910	0.926	0.556
		VB-2	0.806			
Risk barrier (RB)		RB-1	0.827	0.810	0.898	0.647
		RB-2	0.733			
		RB-3	0.753			
		RB-4	0.754			
Tradition barrier (TB)		TB-1	0.756	0.798	0.884	0.685
		TB-2	0.771			
		TB-3	0.775			
		TB-4	0.815			
		TB-5	0.875			
Image barrier (IB)		IB-1	0.788	0.855	0.945	0.689
		IB-2	0.814			
Attitude toward existing product		ATEP-1	0.762	0.750	0.841	0.570
		ATEP-2	0.755			
		ATEP−3	0.800			
Motivation		MOT-1	0.785	0.707	0.818	0.631
		MOT-2	0.896			
		MOT-3	0.805			
		MOT-4	0.772			
Social influence		SI-1	0.853	0.747	0.840	0.572
		SI-2	0.770			
		SI-3	0.810			
		SI-4	0.789			
		SI-5	0.857			
Emotions		EM-1	0.788	0.880	0.904	0.603
		EM-2	0.817			
		EM-3	0.738			
		EM-4	0.775			
		EM-5	0.704			
		EM-6	0.751			
	**Customer characteristics**	Attitude toward existing product	0.743	0.879	0.898	0.543
		Motivation	0.758			
		Social Influence	0.766			
		Emotions	0.842			
Customer resistance to innovation		CRI-1	0.807	0.837	0.912	0.612
		CRI-2	0.855			
		CRI-3	0.838			
		CRI-4	0.831			
		CRI-5	0.797			
		CRI-6	0.788			
		CRI-7	0.8841			

CA, Cronbach's Alpha; CR, composite reliability; AVE, average variance extracted.

Two methods were used with regard to discriminant validity: Fornell–Larcker and Heterotrait-Monotrait ratio (HTMT). No issues were revealed using Fornell–Larcker's method. Every construct's AVE was larger than the variance shared by each construct with the other latent variables (Hair et al., 2017) (see Table 3). The HTMT ratio of correlations was based on the Multitrait–Multimethod Matrix (Henseler and Sarstedt, 2013). As a result, when the HTMT value is more than 0.85, there is an issue with discriminant validity based on these criteria (Kline, 2010). Table 4 shows that the HTMT values are all below the threshold of 0.85, thus confirming the discriminant validity of each pair of constructs (Kline, 2010; Henseler and Sarstedt, 2013).

**Table 3:** Descriptive statistics, correlation matrix, and discriminant validity *via* Fornell and Larcher.

**Constructs**	**Mean**	**SD**	**1**	**2**	**3**	**4**	**5**	**6**
1. Value barrier (VB)	3.855	0.585	**0.778**					
2. Risk barrier (RB)	3.792	0.551	0.602	**0.713**				
3. Tradition barrier (TB)	4.168	0.637	0.432	0.514	**0.829**			
4. Image barrier (IB)	4.502	0.519	0.134	0.165	0.483	**0.809**		
5. Customer characteristics	4.175	0.549	0.335	0.583	0.472	0.305	**0.742**	
6. Customer resistance to innovation	4.133	0.521	0.408	0.574	0.555	0.248	0.602	**0.717**

S.D., standard deviation; n.a, not applicable. Bold values on the diagonal in the correlation matrix are square roots of AVE (variance shared between the constructs and their respective measures). Off-diagonal elements below the diagonal are correlations among the constructs.

**Table 4:** Discriminant validity *via* HTMT.

**Constructs**	**1**	**2**	**3**	**4**	**5**	**6**
1. Value barrier (VB)						
2. Risk barrier (RB)	0.454					
3. Tradition barrier (TB)	0.399	0.679				
4. Image barrier (IB)	0.527	0.604	0.529			
5. Customer characteristics	0.189	0.264	0.359	0.558		
6. Customer resistance to innovation	0.684	0.633	0.667	0.593	0.199	

HTMT should be lower than 0.85.

### Structural model: Hypotheses testing

In this study, the model comprises the direct hypotheses from H_1_ to H_4_ that are described in this section. The hypothesis testing provided the first indication of the direct effect (H_1_), namely, that VB significantly predicted consumer resistance to innovation. Hence, H_1_ was accepted with values of β = 0.366, *t* = 6.467, and *p* < 0.000. The second direct effect (H_2_) of the relationship between RB and consumer resistance to innovation was positively significant, with values of β = 0.174, *t* = 3.019, and *p* < 0.001. Similarly, for H_3_, TB was significantly related to consumer resistance to innovation, so that β = 0.288, *t* = 3.990, and *p* < 0.000. Finally, IB also positively influenced consumer resistance to innovation, so that β = 0.311, *t* = 4.881, and *p* < 0.000. The results are shown in Table 5.

**Table 5:** Structural path analysis: direct effect.

						**Bias and corrected bootstrap**			
						**95% CI**			

**Hypothesis**	**Relationship**	**SB**	**SD**	***t*** **-value**	***p*** **-values**	**(Lower level; upper level)**	**Decision**	***f** ^2^*	**VIF**
H-1	Value barrier (VB)-> customer resistance to innovation	0.366	0.060	6.467	0.000	(0.275; 0.475)	Supported	0.178	1.664
H-2	Risk barrier (RB) -> customer resistance to innovation	0.174	0.066	3.019	0.001	(0.075; 0.293)	Supported	0.035	2.275
H-3	Tradition barrier (TB) -> customer resistance to innovation	0.288	0.070	3.990	0.000	(0.166; 0.404)	Supported	0.087	1.834
H-4	Image barrier (IB) -> customer resistance to innovation	0.311	0.062	4.881	0.000	(0.187; 0.396)	Supported	0.085	1.359

VIF, variance inflation factor.

**Figure 1:**
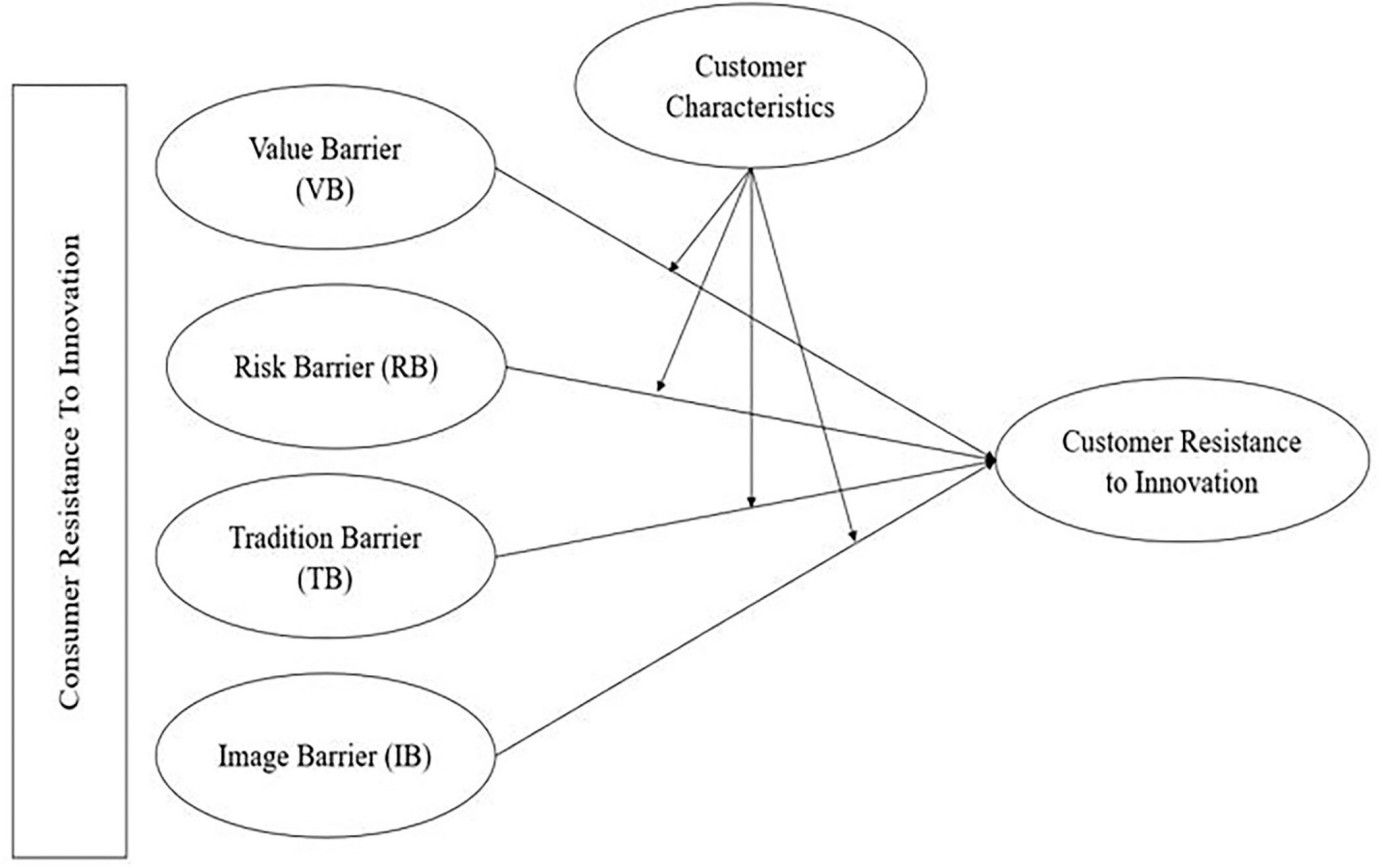
Research framework.

According to the main goals of this study, the moderation test was the key contributor to determining whether consumer characteristics moderate the relationship between the independent elements (i.e., VB, RB, TB, and IB) and the dependent variable (i.e., consumer resistance to innovation) (Figure 1). Therefore, the first interaction, between VB and consumer characteristics toward consumer resistance to innovation, showed a significant interaction, such that β = 0.142, *t* = 4.640, and *p* < 0.000. Therefore, H_5_ is supported. For the second interaction, the relationship between RB and consumer characteristics was insignificant, as the statistical analysis displayed β = 0.045, *t* = 1.024, and *p* < 0.153. As such, H_6_ is not supported. The third interaction, between TB and consumer characteristics toward the consumer resistance to innovation, showed a substantial interaction, with values of β = 0.232, *t* = 3.166, and *p* < 0.001. Therefore, H_7_ is accepted. The final interaction presented the relationship between IB and consumer characteristics toward consumer resistance to innovation. The statistical analysis revealed a positive interaction with values of β = 0.158, *t* = 2.988, and *p* < 0.000. Therefore, H_8_ is supported. Table 6 displays all the results described above.

**Table 6:** Structural path analysis: the interaction effect (moderation).

						**Bias and corrected bootstrap**			
						**95% CI**			

**Hypothesis**	**Relationship**	**SB**	**SD**	***t*** **-value**	***p*** **-values**	**(Lower level; upper level)**	**Decision**	***f** ^2^*	**VIF**
H-5	VB X CH->CRIN	0.142	0.052	4.640	0.000	(0.041; 0.203)	Supported	0.026	1.148
H-6	RB X CH-> CRIN	0.045	0.044	1.024	0.153	(−0.117; 0.020)	Not supported	0.002	1.568
H-7	TB X CH->CRIN	0.232	0.046	3.166	0.001	(0.027;0.176)	Supported	0.018	1.072
H-8	IB X CH-> CRIN	0.158	0.050	2.988	0.000	(0.064; 0.232)	Supported	0.039	1.471

VIF, variance inflation factor.

VB X CH->CRIN, Value Barrier X Consumers' Characteristics->Customer Resistance to Innovation; RB X CH-> CRIN, Risk Barrier X Consumers' Characteristics->Customer Resistance to Innovation; TB X CH->CRIN, Tradition Barrier X Consumers' Characteristics-> Customer Resistance to Innovation; IB X CH-> CRIN, Image Barrier X Consumers' Characteristics-> Customer Resistance to Innovation.

**Figure 2:**
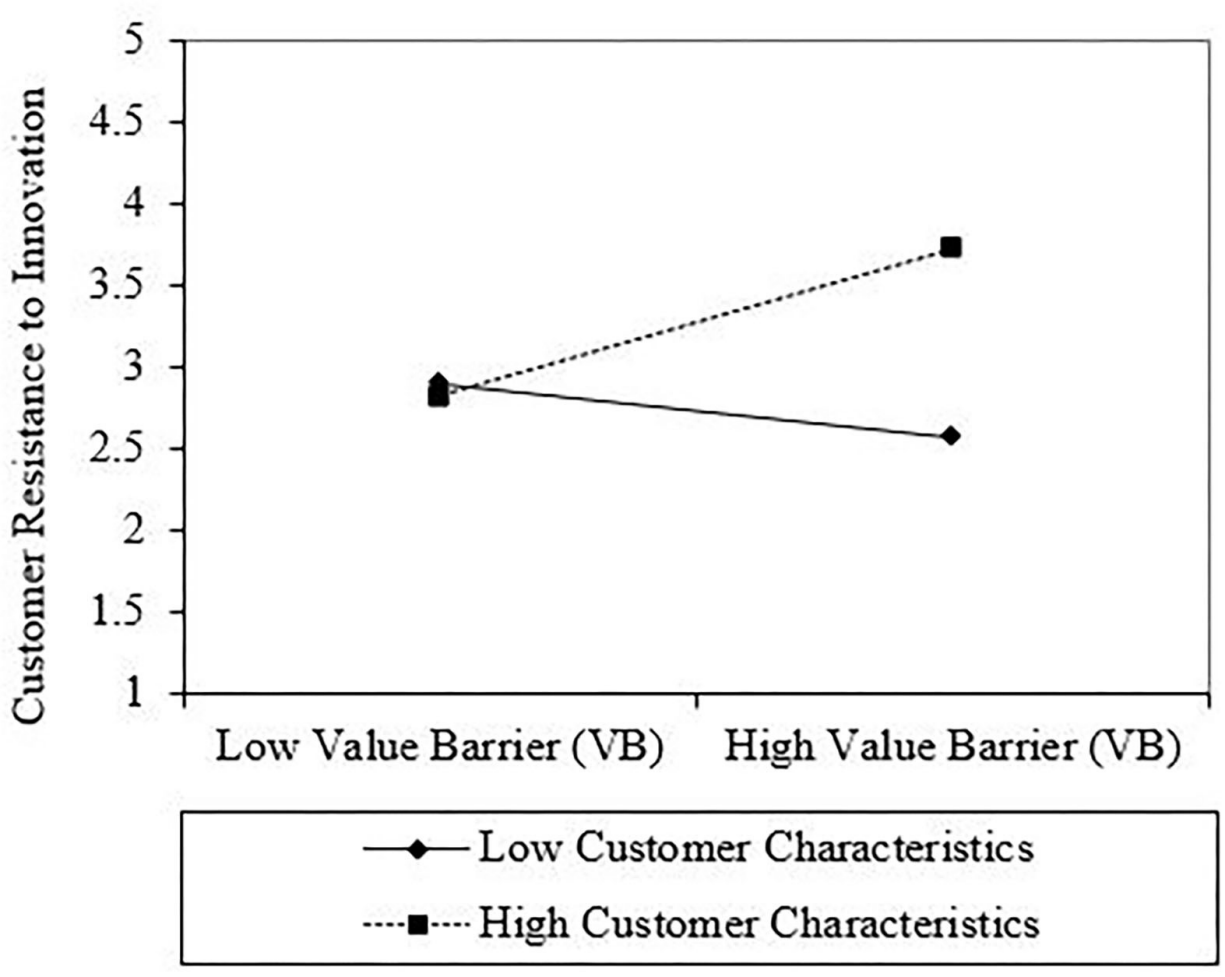
Interaction plot of value barrier (VB) and customer characteristics interaction on the customer resistance to innovation.

**Figure 3:**
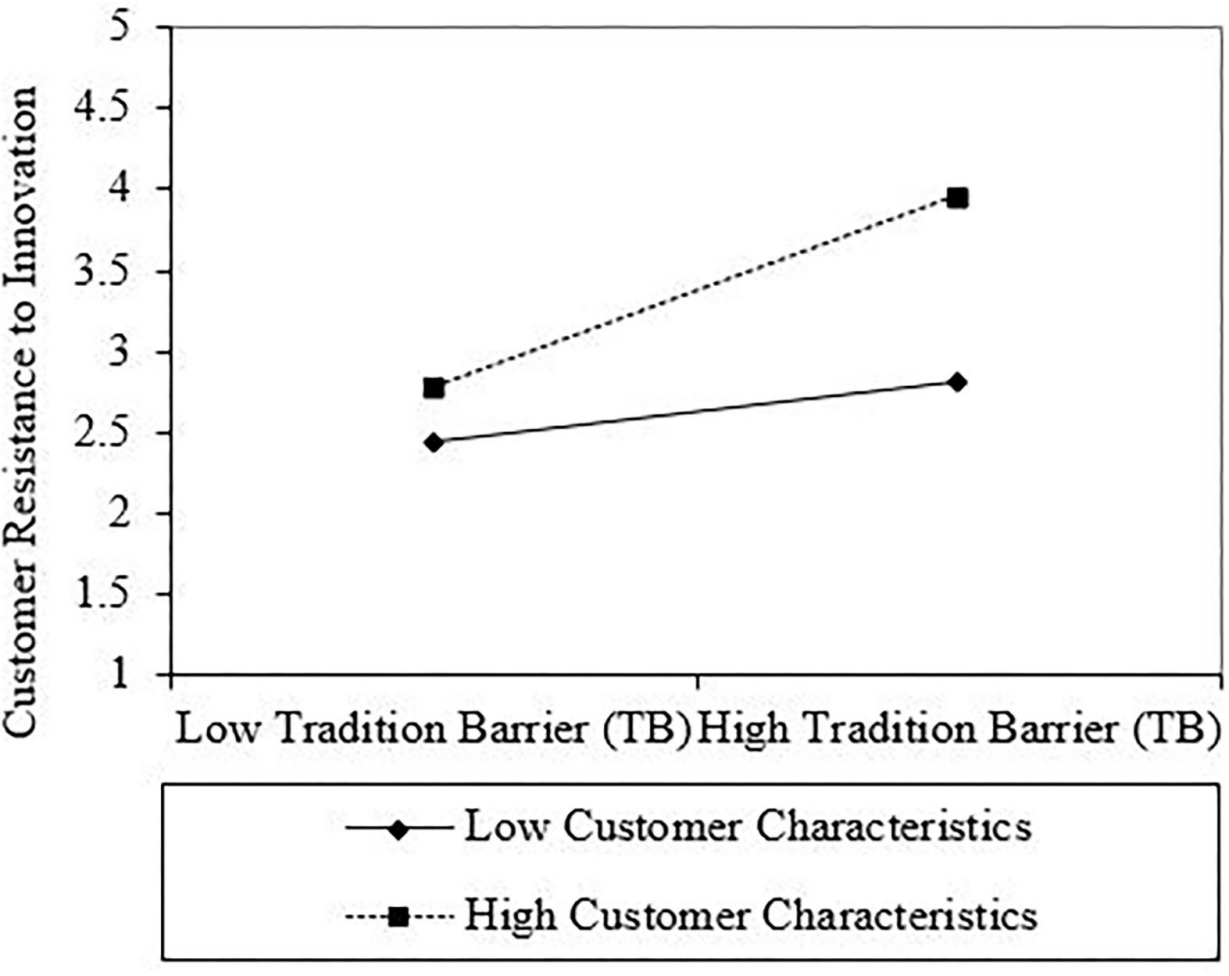
Interaction plot of tradition barrier (TB) and customer characteristics interaction on the customer resistance to innovation.

**Figure 4:**
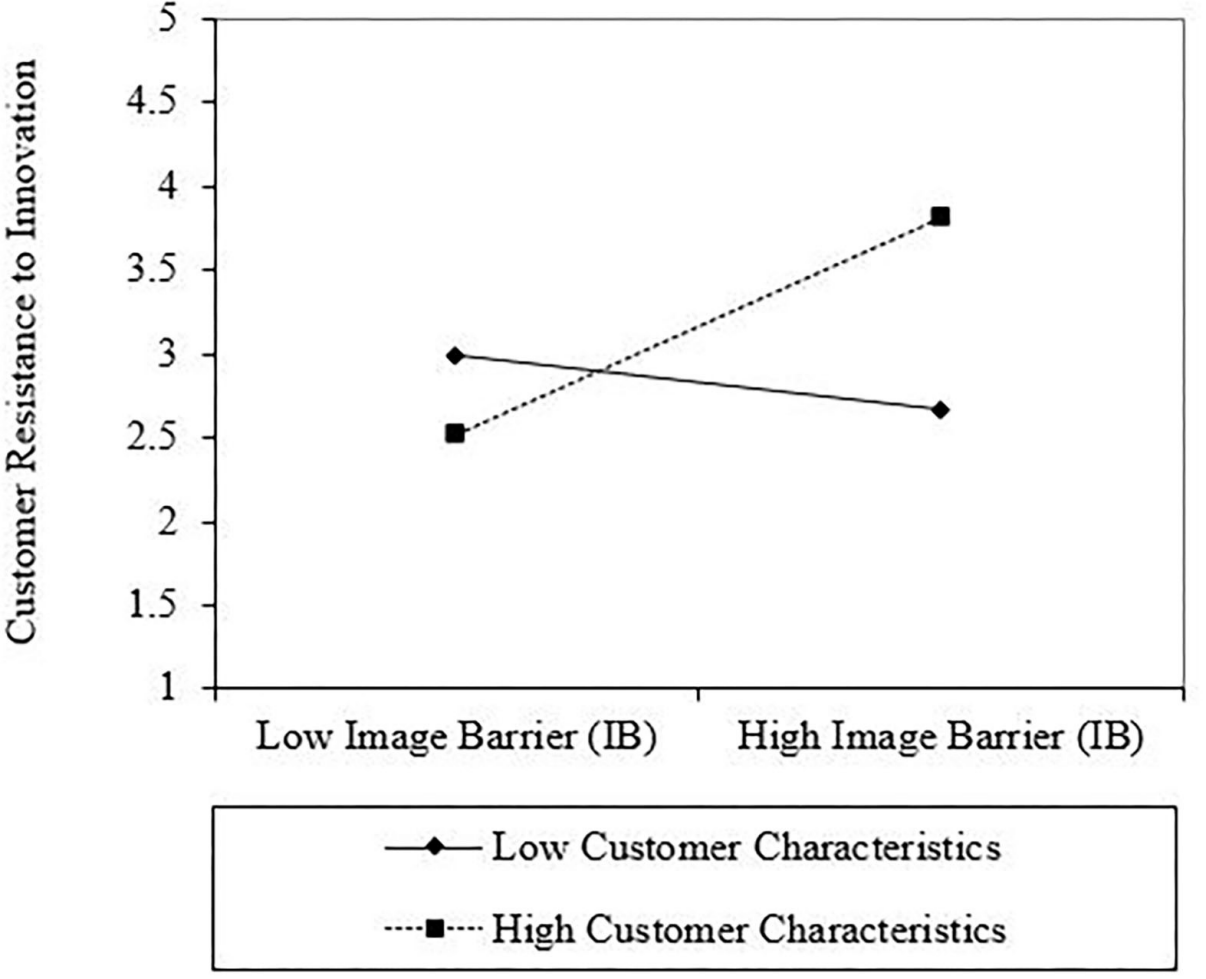
Interaction plot of tradition barrier (IB) and customer characteristics interaction on the customer resistance to innovation.

According to Dawson (2014), this may be followed by an interaction plot. As a result, to examine the gradient of the slopes, this study used an interaction plot for all interactions. As shown in Figure 2, the line labeled “High Consumer Characteristics” for the first interaction has a steeper gradient if contrasted with “Low Consumer Characteristics”, which indicates that when consumer characteristics are higher, the positive relationship between VB and consumer resistance to innovation is stronger (see Figure 2). The second interaction, between TB and consumer characteristics toward consumer resistance to innovation, showed that the positive relationship between TB and consumer resistance to innovation is greater when consumer characteristics are higher rather than lower (see Figure 3). Figure 4 presents the interaction between IB and consumer characteristics on consumer resistance to innovation. As can be seen from the interaction, the consumer characteristics value strengthens the positive relationship between IB and consumer resistance to innovation, such that the relationship is stronger when the consumer characteristics are higher (see Figure 4).

In terms of the overall explanatory power of the model, *R*^2^ = *0.571* for consumer resistance to innovation can be classified as having a moderate-to-substantial effect (Hair et al., 2017). In addition, the Stone–Geisser blindfolding sample reuse technique reveals *Q*^2^ values larger than zero, thus indicating that the research model in this study is good for predicting consumer resistance to innovation (*Q*^2^ = *0.211*) (Hair et al., 2017). Finally, with respect to the overall goodness-of-fit (GoF), the residual index of the standardized root mean square (SRMR) yielded a value of 0.042, which was significantly below the 0.08 cutoff (Henseler and Sarstedt, 2013). Likewise, SRMR's 95% bootstrap quantile is 0.059 and is, therefore, higher than the SRMR value (0.042), indicating that the model possesses a good fit (Hair et al., 2017). Lastly, the discrepancy indexes unweighted least squares discrepancy (dULS) and geodesic discrepancy (dG) are also below the bootstrap-based 95% percentile (dULS = 1.371 < HI 95 of dULS = 2.852; dG = 0.462 < HI 95 of dG = 0.881) (Hair et al., 2017). Therefore, the discrepancy between the model-implied correlation matrix and the empirical study is not significant, thus suggesting that there is no reason to reject the model and that the model is likely to be true (Henseler, 2017).

## Discussion

Based on the results for hypothesis H1, VB significantly predicts consumer resistance to innovation. The second direct effect, H2, of the relationship between RB and consumer resistance to innovation was positively significant. Similarly, for H3, TB was significantly related to consumer resistance to innovation. IB also positively influenced consumer resistance to innovation H4 was supported.

In regard to the interactions, the effect found that H5 is supported where the interaction between value barrier and consumers' characteristics was significantly related to customer resistance to innovation, thus H5 is supported. The second interaction between the risk barrier and consumers' characteristics were insignificant; as the statistical analysis indicated, H6 is not supported. The third interaction, between TB and consumer characteristics toward consumer resistance to innovation, revealed a significant interaction. Therefore, H7 is confirmed. The final interaction presents the relationship between IB and consumer characteristics toward consumer resistance to innovation. The statistical analysis showed a positive interaction; therefore, H8 is supported. Above all, most of the variables are true predictors of consumer resistance to innovation.

This study makes substantial contributions to the existing literature by expanding the set of positive outcomes to be expected among factors (i.e., value barrier, risk barrier, tradition barrier, and image barrier) and resistance to innovation in a low-income population. Thus, this study makes a critical contribution by examining these elements in a single research model. Importantly, this tries to extend the theory by investigating the moderating roles of consumer characteristics on the relationships among value barrier, risk barrier, tradition barrier, image barrier, and resistance to innovation. Consumer characteristics greatly influence the demand and uptake of innovations, especially digital innovations (Oh et al., 2022).

Productivity applications in the education industry are among the best tools for managing tasks. This is because they give users access to both personal and educational digital resources. However, productivity applications also face challenges in the educational setting, such as consumer resistance to innovation (Al Halbusi et al., 2020). As a result, this research has several implications based on this study's findings that can be useful in assisting students and universities in increasing the adoption of productivity applications. It also provides deep insight into the factors that significantly influence low-income students' resistance to innovation (Al-Gahtani, 2003).

The insights that can be gained from these results are of vital importance to research on resistance to innovation among low-income populations and for policymakers. Being of low income invites certain conditions that might affect how and when innovations might be resisted. Policy interventions, for instance, might be designed to address the issues revealed by the results of this study. The significance of these results and their use to help overcome resistance to innovation are vital not only to the targeted population group but also to national economic imperatives. The competitiveness of nations and businesses hinges greatly on their ability to generate value through utilizing digital innovations. In contrast, economic competitiveness and the welfare benefits of digital innovations are greater when nations and businesses diverge significantly from utilizing cutting-edge technology (Curzi et al., 2019; El-Haddadeh, 2020). Research such as this on resistance to innovation, especially among underresearched populations, is of vital importance not only to scholars but also to policymakers.

## Limitations and suggestions for future research

Just as there are limitations in any research, this study has several limitations. First, because students were the sample used to collect data, the findings cannot be applied to a nonstudent population. As a consequence, future studies should evaluate the sample size, including individuals who are not students, for the findings to be more generally applicable. Moreover, the majority of the respondents were between the ages of 20 and 30. This again limits the generalizability of the results. Thus, it is recommended that future studies encompass a broader age range. This survey, on the other hand, solely includes students from public universities. Future research should include students from both public and private universities in Saudi Arabia. The next limitation of this study relates to the fact that it was a cross-sectional study. As noted, a cross-sectional survey design limits the researcher's ability to identify causality. In other words, a cross-sectional survey design deduces a single data collection point, which is not sufficient to pinpoint causal relations. Thus, the quantitative cross-sectional design aims to identify the mutual occurrence that links antecedents and consequences instead of identifying causality. Thus, since the data that relied on the analysis of the current study were cross-sectional, a longitudinal approach is recommended for future research to provide a better position for researchers to draw causal conclusions, as the Kingdom of Saudi Arabia is huge and has a good number of public universities to study. Lastly, as mentioned previously, most studies on resistance to innovation and its consequences were conducted in the United States and European countries, and there was a need to establish this kind of research in Middle Eastern countries. This is why this study was conducted in Saudi Arabia. As this study is limited to Saudi universities, however, the results cannot be generalized to other Middle Eastern countries due to geographical, political, cultural, and other differences. Therefore, it is recommended to do the same research in other Middle Eastern countries (Kuwait, UAE, Bahrain, Oman, and others), which may enable generalization of the existing findings.

## Conclusion

The purpose of this study was to investigate the factors that influence low-income populations' resistance to innovation. Specifically, the case of undergraduate students on financial support and resistance to utilizing digital productivity applications was examined. This study constructed a model of consumer resistance to innovation based on resistance to innovation theory to analyze the variables affecting consumer resistance to innovation (Arnold, 1960; Ram and Sheth, 1989). This framework and the results of this study help to provide a comprehensive understanding of the variables influencing low-income consumer resistance to innovation. This research sheds light on a unique and largely overlooked consumer segment, namely, low-income students, and its findings can be applicable to other low-income population segments. The results of this study reveal a greater need to explore this topic further. For example, it has been identified that low-income populations might be perceived as having a lower value and thus suffer from both a value barrier and a tradition barrier. There remains much to unravel regarding how and why such populations might perceive these barriers differently. Future research could explore these barriers and conduct a comparative study to examine how low-income and high-income students might perceive these barriers differently regarding the same digital innovations. Learning about such issues will greatly inform policy design and interventions to help bring the benefits of such innovations to low-income populations.

## Data availability statement

The raw data supporting the conclusions of this article will be made available by the authors, without undue reservation.

## Ethics statement

The studies involving human participants were reviewed and approved by University of Hail. Written informed consent for participation was not required for this study in accordance with the national legislation and the institutional requirements.

## Author contributions

MAl contributed to research problem formulation, study design, data collection, and overall write up. HAH contributed to questionnaire design, data analysis, and results section write up. MAb contributed to the literature review and discussion section write up. HA contributed to implications, conclusion sections, final proofreading, and quality checks. All authors contributed to the article and approved the submitted version.

## Conflict of interest

The authors declare that the research was conducted in the absence of any commercial or financial relationships that could be construed as a potential conflict of interest.

## Publisher's note

All claims expressed in this article are solely those of the authors and do not necessarily represent those of their affiliated organizations, or those of the publisher, the editors and the reviewers. Any product that may be evaluated in this article, or claim that may be made by its manufacturer, is not guaranteed or endorsed by the publisher.
